# Long noncoding RNAs and neuroblastoma

**DOI:** 10.18632/oncotarget.4251

**Published:** 2015-06-10

**Authors:** Gaurav Kumar Pandey, Chandrasekhar Kanduri

**Affiliations:** ^1^ Department of Medical Genetics, Institute of Biomedicine, Sahlgrenska Academy, University of Gothenburg, Gothenburg, Sweden

**Keywords:** noncoding RNA, neuroblastoma, MYCN, NBAT1, neuronal differentiation

## Abstract

Neuroblastoma is a disease that affects infants and despite intense multimodal therapy, high-risk patients have low survival rates (<50%). In recent years long noncoding RNAs (lncRNAs) have become the cutting edge of cancer research with inroads made in understanding their roles in multiple cancer types, including prostate and breast cancers. The roles of lncRNAs in neuroblastoma have just begun to be elucidated. This review summarises where we are with regards to lncRNAs in neuroblastoma. The known mechanistic roles of lncRNAs during neuroblastoma pathogenesis are discussed, as well as the relationship between lncRNA expression and the differentiation capacity of neuroblastoma cells. We speculate about the use of some of these lncRNAs, such as those mapping to the 6p22 hotspot, as biomarkers for neuroblastoma prognosis and treatment. This novel way of thinking about both neuroblastoma and lncRNAs brings a new perspective to the prognosis and treatment of high-risk patients.

## INTRODUCTION

Neuroblastomas (NBs) are the third most common tumour affecting infants and young children, after leukemia and brain/central nervous system tumours. Moreover, around 7% of the total tumours observed in children are NBs. It is a disease of the sympathetic nervous system believed to arise due to improper development and differentiation of the neural crest cells [1, 2]. The most distinguishing feature of NBs that makes them challenging from a clinical and research perspective is the degree of heterogeneity observed within their several subtypes. Multiple factors such as age of the patient at diagnosis, stage of the disease and genetic profile of the tumour, along with other molecular characteristics determine the clinical outcome of the disease. These outcomes can be very different between NB subtypes ranging from low-risk regressive to high-risk aggressive tumours. The low-risk NBs are characterized by tumours with little or no risk, favourable clinical prognosis, spontaneous regression of the tumours and numerical changes in chromosomal copy numbers (INSS stages 1, 2 and 4S). High-risk NBs, on the other hand, are highly aggressive, metastatic tumours with poor prognosis, low or no response to chemotherapy and show clinically unfavourable outcomes (INSS stages 3 and 4) [3, 4].

Over the years, genetic and clinical research has established characteristic chromosomal alterations as markers for a poor clinical outcome in high-risk NB patients. One of the earliest identified genetic alterations in high-risk NBs was focal amplification at the chromosomal location 2p24, which harbours the oncogene *MYCN* [5–8]. Amplification of this oncogene is observed in around 20–25% of NB cases and is associated with an unfavourable outcome. Additional genetic lesions have also been reported from other chromosomes. These include deletions at chromosome 1p, 3p, 11q and gains at 1q, 2p and 17q regions and they are observed in around 50% of the NB cases [9–13]. Apart from these major genetic aberrations in NB patients, genome wide studies have also focussed on identifying risk associated mutations. However, there appears to be a paucity of mutations in NB tumours barring a few exceptions, such as activating mutations in the *ALK* gene. Additionally, several predisposing single-nucleotide polymorphisms (SNPs) are present in coding as well as noncoding regions of the genome, in NB patients. These include high-risk associated SNPs present in the intron of lncRNAs, *NBAT1* (also known as *CASC14 or NBAT-1*) and *LINC00340 (CASC15)*, or adjacent to protein coding genes, *LMO1*, *LIN28B* and *BARD1* [14, 15]. Some of these SNPs have been well studied, such as the ones included in *NBAT1* and *LIN28B*, and their role in NB pathogenesis further elucidated [16, 17].

Despite overall genetic and molecular knowledge of NBs, we have a very limited understanding of how these features contribute to the molecular events resulting in aggressive NBs. Also, it is essential to underline that the average percentage of patients who survive high-risk NB is just 50% [18]. This underscores the need for identifying new players and biomarkers for better risk stratification, as well as gaining further insights into the underlying biology of NB tumours. One of the prospective players that could significantly contribute towards deeper understanding of NB tumours is a molecule known as long non coding RNA (lncRNA). These lncRNA (more than 200bp in length) species have been recognized lately as integral components of the eukaryotic regulatory gene network [19–21]. A significant number of noncoding RNAs including lncRNAs have been functionally implicated in a wide variety of cellular processes [22–25] and their aberrant expression has been shown to be one of underlying causes for tumor initiation and progression in diverse cancer types. For example, lncRNAs such as *HOTAIR* (breast cancer), *NKILA* (breast cancer metastasis), *NBAT1* (neuroblastoma), *MALAT1* (lung, breast, prostate and cervix cancer), *MDC1-AS* (bladder cancer), *lncRNA-886* (esophageal and gastric cancers) and *PCAT-1* (prostate cancer) have been implicated in tumorigenesis [17, 21, 26–33]. Many of these lncRNAs have been shown to regulate crucial biological processes such as cell proliferation, invasion and metastasis during development of cancer [34–37].

Similar to other human cancers, it is reported that several lncRNAs are being deregulated during NB pathogenesis (Table 1) [17, 38–40]. In the present review, we provide an overview of NB tumours from an lncRNA perspective. We discuss the underlying molecular connections between lncRNA expression and the observed tumorigenic properties of NBs. Furthermore, as NB is a differentiation disorder, we reflect on how lncRNAs could contribute to NB pathogenesis as newly recognized regulatory molecules governing lineage specific differentiation mechanisms. Lastly, we discuss whether lncRNAs, similar to their protein counterparts, could be utilized as biomarkers for stratifying and assessing the risks associated with the disease.

**Table 1:** Deregulated long noncoding RNAs in neuroblastoma tumors

Name	Description	Biological Function
*T-UC.300A*	Decreased expression upon RA induced differentiation of SH-SY5Y cells.	Oncogenic properties with pro-proliferation and invasive properties (48).
*ncRAN*	Maps to 17q gain region, its overexpression significantly associated with poor prognosis.	Possess oncogenic properties. Depletion results in decreased proliferation capacity of NB cells (52).
*lncUSMycN*	Maps to 2p amplified region, higher expression can independently predict poor outcome in NB patients.	Post-transcriptionally regulates expression of *MYCN* through association with RNA binding protein NonO (40).
*linc00467*	*MYCN* target gene.	Increases survival of NB cells by repressing *DKK1* (58).
*NDM29*	Maps to frequently deleted 11p15.3 region.	Overexpression in NB cell lines leads to decreased proliferation, differentiation and increased chemosensitivity (39).
*CAI2*	Located in intron 2 of the *CDKN2A* gene. Higher expression in high-risk patients.	Not characterized/Unknown (57).
*NBAT1*	Maps to 6p22 locus, which harbors disease associated SNPs. Lower expression is associated with poor outcome in NB patients.	Suppresses neuroblastoma cell proliferation and invasion by epigenetically repressing target genes, and promotes neuronal differentiation by repressing *NRSF*/*REST* (17).
*Pauppar*	It is located upstream of neural transcription factor *Pax6*.	Complex transcriptional regulation of neural genes via associating with neural transcription factor *Pax6* (63).

### Understanding the etiology of neuroblastoma through deregulated lncRNAs

There has been extensive lncRNA research in the last decade thanks to innovative high throughput approaches and the use of robust analysis pipelines. According to the latest estimates, there are about 58, 648 annotated lncRNAs present in the human genome [41]. They map to intergenic as well as intronic regions of the genome and can be present in an antisense orientation to the adjacent protein-coding gene. With the passing of time, greater patho-physiological roles of these regulatory molecules are being uncovered. They can act as a scaffold for the recruitment of chromatin modifying enzymes such as PRC2 and SWI/SNF complexes and regulate local or distant gene expression [42]. LncRNAs can modulate the signalling pathways of tumour suppressor or oncogenes by limiting or activating their expression [26]. In addition they can serve as sponges for miRNA or proteins for exhibiting their regulatory function [43].

In the current section, we discuss the published literature about lncRNAs reported in NB tumours and overview their functional contribution towards disease etiology. In addition, we suggest future investigations to further strengthen the connection between lncRNAs and NB pathogenesis.

#### T-UCRs

Transcribed ultra-conserved regions (*T-UCRs*) are defined as a group of lncRNAs larger than 200 base pairs in length and show 100% conservation between human, rat and mouse genomes. 481 such regions were reported for the first time in the study carried out by *Bejarno et al* in 2004 [44]. The highest degree of conservation among these noncoding regions can be interpreted as having a putative functional role and a plausible deleterious effect when deregulated in human diseases. Indeed, *Calin et al* found aberrant expression of 88 (9%) of the 962 *T-UCR* probes covering 481 *T-UCRs* in leukaemia and hepatocellular carcinomas, establishing their plausible role in carcinogenesis [45]. These observations have been extended to other human cancers, including NBs, to further evaluate their clinical significance in patient prognosis and cancer progression.

*Scaruffi et al* evaluated the expression of 481 *T-UCRs* in 34 high-risk, aggressive NB patients using qRT-PCR [46]. Of these, 28 *T-UCRs* were found to be significantly associated with patient prognosis. Interestingly, the authors applied a prediction model where a combination of *T-UCR* expression information was used to accurately predict clinical outcomes. An expression signature of 15 upregulated *T-UCRs* was able to distinguish long and short-term survivors with a high degree of sensitivity and specificity. Furthermore, these multi-*T-UCR* signatures could independently predict the event free survival (EFS) and overall survival (OS) in this small cohort of high-risk NBs. The authors report an inverse correlation of 9 *T-UCRs* with 5 complementary miRNAs suggesting a perturbed miRNA/*T-UCR* network as one of the plausible factors in NB pathogenesis.

Another parallel study by *Mestdagh et al* has analysed the expression profile of all 481 *T-UCRs* in NB and assigned their putative functions using multi-level transcriptomic data with respect to genomic location and coregulation with the host region and surrounding regions [47]. 237 *T-UCRs* are expressed independently of the host or nearby protein coding genes; however all of them positively correlate to the expression pattern of the corresponding flanking genes. Using qRT-PCR analysis in a cohort of 49 NB tumours, this study found 7 *T-UCRs* to be overexpressed in *MYCN* amplified tumours relative to the non-amplified ones. Furthermore, three of these *T-UCRs* (*uc.279, uc.364* and *uc.460*) show a positive response to *MYCN* gene along with a similar expression profile in a larger patient dataset (366 samples). The authors utilized a functional genomics approach to show that *T-UCRs* are linked to carcinogenic pathways such as cell proliferation, differentiation, apoptosis, cell cycle, DNA repair and replication.

A more recent study explored the functional role of *T-UCRs* during neuronal differentiation [48]. Using a tilling array to profile the expression of 481 *T-UCRs*, it was reported that 32 *T-UCRs* showed significant differential expression during retinoic acid (RA) induced neural differentiation of NB cell lines. Furthermore, downregulation of one of the intergenic transcripts *T-UC.300A* resulted in a significant decrease in the proliferative and invasive capacities of NB cells. As this transcript has a distinct decreased expression in differentiated NB cells and a phenotype similar to an oncogene, it is possible that overexpression of *T-UC.300A* may contribute to NB pathogenesis. It would be interesting to see how many of these differentially expressed *T-UCRs*, during neuronal differentiation, could be linked to NB pathogenesis. The examples discussed above present an interesting case favoring a greater role for *T-UCRs* in NB pathogenesis; however the significance of their differential expression remains to be fully explored. It is important to systematically evaluate their clinical significance in larger and more diverse patient datasets. Further studies on their functional characterization and identification of deregulated downstream effectors/pathways, due to perturbed *T-UCR* expression, will contribute towards their future development as molecules of interest in understanding biology of NB tumours, as well as in developing novel intervention strategies.

#### LncRNAs at regions of chromosomal gains

Genomic instability is a characteristic event associated with cancerous cells resulting in either a gain or loss of specific chromosomal regions. These genetic changes provide tumour cells with specific survival advantages and adversely affect the patient's health. In NBs, distinct chromosomal gains such as the gain of 17q, 1q and 7q chromosome arms are frequently observed in high-risk NB patients. Out of these, the 17q gain is the most frequent alteration associated with a poor outcome [49]. This chromosomal region harbours protein coding genes such as *Survivin* and *PPM1D* which are candidates known for their role in NB aggressiveness [50, 51]. Similarly, these chromosomal regions are also inhabited by several lncRNAs whose transcriptional output might have a role in disease phenotype.

*Yu et al* had hybridized 236 NB tumours on a cDNA microarray to identify candidate lncRNAs with significant differential expression in patients with the 17q gain [52]. The authors found a previously unreported lncRNA, a non-coding RNA expressed in aggressive NBs (*ncRAN*). As the name suggests, *ncRAN* shows significant overexpression in NBs with the 17q gain, known to be associated with a poor prognosis. In addition, the Multivariate Cox proportional hazard model found that *ncRAN* could act as independent prognostic factor. Functional characterization of *ncRAN* involving depletion or overexpression in NB cell lines suggests that *ncRAN* possesses oncogenic properties. These clinical and *in vitro* functional data indicate that overexpression of *ncRAN* lncRNA could be one of the many contributing factors involved in the aggressiveness of the high-risk NBs with a 17q gain. However, mechanistic insights into the exact role of *ncRAN* in NB pathogenesis are still awaited. These results also support the hypothesis that overexpression of lncRNAs caused due to genetic instability could significantly contribute to NB progression. Thus it would be worth identifying such overexpressed lncRNAs and characterizing the consequences of their increased transcriptional dosage in NB pathogenesis.

#### Focal amplification at 2p24 is a reservoir of potential oncogenes

It has been 30 years since the amplification at the chromosomal region 2p24 was observed in NB patients and it was found to be associated with poor prognosis [5, 53]. This amplification can be 1Mb in size, inhabiting many genes (both coding and noncoding) that could potentially contribute to the aggressive phenotype in the disease. Since then, research has been oriented to identify potential candidates in this amplicon and evaluating their prognostic value in NB pathogenesis. One of the most studied genes that maps to this genetic locus is *MYCN* and its genetic amplification is observed in around 25–30% NB patients. Although the *MYCN* gene has been well characterized for its role as an oncogene in NB pathogenesis, the intriguing possibility remains whether co-amplified lncRNAs along with *MYCN* could additionally contribute to NB phenotype and thus could serve as potential biomarkers or drug targets. Therefore, it would be fascinating to investigate their prognostic value and characterize their biological function in NB pathogenesis.

**Figure 1:**
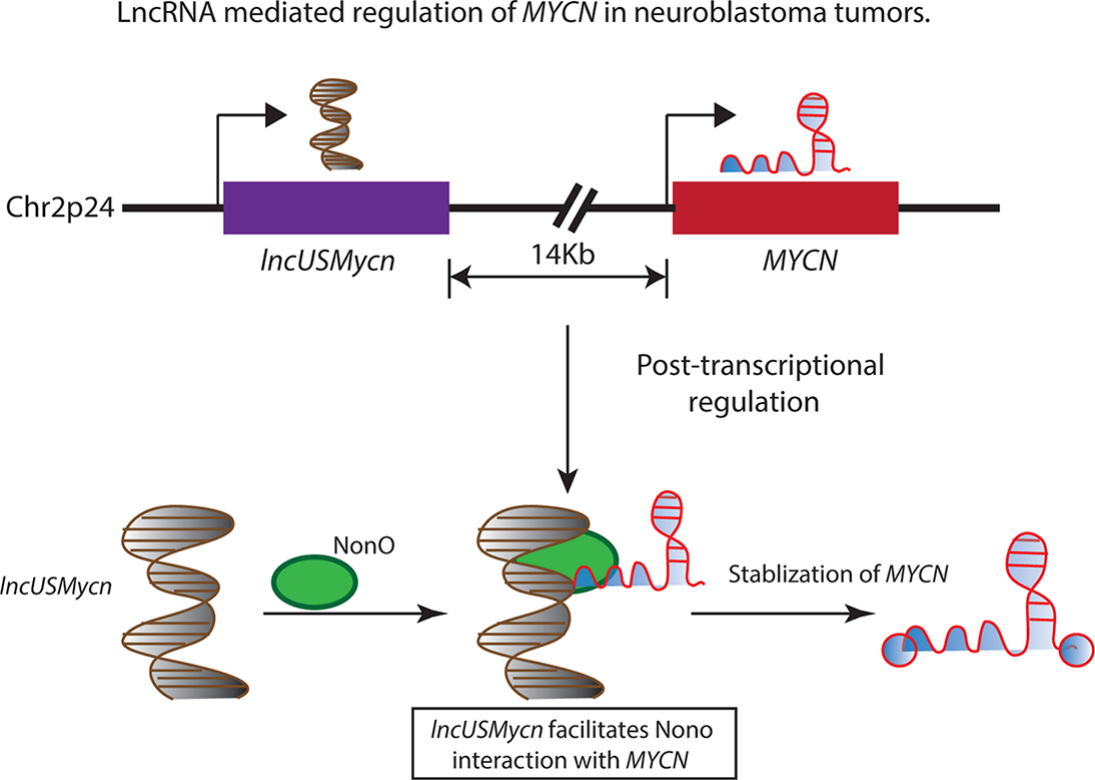
The 2p24 chromosomal region is amplified in several high-risk neuroblastoma patients and is associated with adverse outcomes The amplified region harbors the *MYCN* oncogene and a lncRNA *lncUSMycn*, located 14 kb upstream of the *MYCN* oncogene. The *lncUSMycn* noncoding RNA post-transcriptionally regulates the expression of *MYCN* by acting as a scaffold for the RNA binding protein NonO and facilitating the interaction between NonO and *MYCN*. This RNA-protein interaction leads to the stabilization of the *MYCN* transcript and elevating the *MYCN*-driven transcriptional program in affected individuals.

In line with this hypothesis, *Liu* and colleagues have recently reported a lncRNA 14 kilobase (kb) upstream of the *MYCN* gene, *lncUSMycN*, which maps to the frequently amplifed region on chromosome 2p [40]. *lncUSMycN* is overexpressed in *MYCN* amplified NB cell lines and patients with an amplification of the *MYCN* oncogene. Ablation of its expression using siRNA/shRNA in NB cell line SK-N-BE2 results in decreased expression of *MYCN* mRNA as well as its protein. This regulation of *MYCN* by *lncUSMycN* seems to be dependent on a RNA binding protein NonO, whose interaction with *lncUSMycN* post-transcriptionally regulates *MYCN* expression (Figure 1). Interestingly, NonO also interacts with *MYCN* mRNA. NonO is a RNA binding protein that plays an important role in RNA processing and double-strand break repair. These observations raise several interesting questions. How does the dual interaction of NonO with *MYCN* and *lncUSMycN* influence the post-transcriptional stabilization of *MYCN*? Does *MYCN* mRNA directly bind to NonO or *lncUSMycN* facilitating NonO interaction with *MYCN* mRNA? It is important to mechanistically characterize the significance of these RNA-protein and protein-protein interactions in *MYCN* amplified NBs to clearly understand the effect of *lncUSMycN* overexpression on *MYCN* upregulation. Functionally, *lncUSMycN* possesses oncogenic properties as it induces increased proliferation in NB cell lines and its depletion in xenograft models caused a significant reduction in tumour volume. The analysis of the prognostic significance of *lncUSMycN* and *NonO* in NB patients in three independent patient data sets (45 NB tumours, Versteeg and Kocak data sets) revealed that both *lncUSMycN and NonO* are independently associated with poor outcome in NB patients. Similar to *lncUSMycN*, a more recent study by *Pandey et al* has also reported a novel transcript on chromosome arm 2p. This unannotated lncRNA (named as *CUFF121734*) is located 293kb away from the *MYCN* gene and found to be overexpressed in *MYCN* patients [17]. However, its effect on *MYCN* expression or its role in NB pathogenesis remains to be investigated.

Based on these observations, it is tempting to speculate that co-amplified lncRNAs could influence the expression of amplified *MYCN* by utilizing unknown diverse regulatory mechanisms. These results raise a very important question of whether co-amplified lncRNAs on chromosome arm 2p are essential for *MYCN* driven metastatic disease in NB patients. Since *MYCN* is a global transcription factor with diverse biological functions, understanding the functional nexus between lncRNAs and *MYCN* would help in devising specific therapeutic interventions based on lncRNAs, so that these amplified molecules could be used as targets to neutralize the *MYCN* driven transcriptional programme in high-risk NB patients.

Researchers can draw clues from a recent mouse genetic study, which demonstrates that expression of a co-amplified lncRNA *PVT1* is essential for the tumorigenic potential as well as for the overexpression of the co-amplified oncogene *MYC* [54]. Here the lncRNA and MYC act in conjunction towards cancer progression, where the lncRNA regulates the phosphorylation of the MYC protein and protects it from destabilization and degradation. This study implicates lncRNA in the regulation of amplified oncogenes and suggests that similar functional roles for other such co-amplified lncRNAs.

#### Potential tumour suppressor lncRNA at 1p and 11q chromosomal deletions

Regions of allelic deletion on chromosomes 1 and 11 are commonly observed in NB patients with poor prognosis [55, 56]. These chromosomal segments are believed to harbour genes with tumour suppressor properties including protein coding genes as well as lncRNAs. LncRNA Neuroblastoma differentiation marker 29 (*NDM29*) that maps to the 11p15.3 region is reported as frequently deleted in NBs [39]. Cells with overexpression of *NDM29* exhibit onco-suppressive properties both *in vitro* as well as *in vivo*. In addition, these cells show an increased sensitivity to antiblastic drugs, thus making *NDM29* a potential candidate from a therapeutic perspective. With this evidence favouring a plausible role of *NDM29* in containing neuroblastoma progression, it becomes pertinent to evaluate its clinical potential using mouse based genetic studies as well as gain more insights into its regulatory contribution. A recent study also revealed a putative list of 6 such RNAs with one mapping to the 11q region and the rest to 1p proximal region [17]. Functional and clinical characterization of these prospective candidates might yield useful information about their roles in NB etiology.

#### 6p22, harbouring disease associated SNPs and lncRNAs, a major susceptibility locus for NB

Genome wide association studies (GWAS) are typically employed to identify susceptibility alleles in individuals and score for their association with traits such as disease susceptibility. They have revealed many candidate genes with potential roles in carcinogenesis. In NB patients, a GWAS study performed on a large number of patients (1032 NB vs. 2043 control) has identified a high-risk NB associated polymorphism at the chromosomal locus 6p22 [15]. The SNP, *rs6939340* when homozygous for the ‘G’ allele has an increased probability of high-risk neuroblastoma, poor outcome, and decreased event free survival. The location of this SNP is highly interesting from an lncRNA perspective as there are no protein-coding genes present in the vicinity. It is present in the intronic region of two lncRNAs, *NBAT1* (neuroblastoma associated transcript1, *LOC729177 or CASC14)* and *LINC00340* (*CASC15*). However, the functional connection between risk-SNP and these lncRNAs and their plausible role in NB tumors was not known until recently.

Using RNA-seq approach, a recent study has profiled transcriptomes of 15 tumours representing low and high-risk subtypes [17]. Bioinformatical analysis of the sequencing data revealed many annotated as well as non-annotated lncRNAs, including *NBAT1*, as differentially expressed between low and high-risk NB subtypes. Expression analysis of *NBAT1* in two independent cohorts (93 and 498) of NB tumours revealed that patients with lower expression of *NBAT1* are associated with poor clinical outcome and decreased overall and event free survival. More importantly, *NBAT1* expression levels can independently predict event free survival in NB patients. A strong link between differential *NBAT1* expression and clinical outcome raises the following important questions; 1. What causes decreased expression of *NBAT1* in high-risk NBs? 2. What is the functional role of *NBAT1* in NB pathogenesis?

**Figure 2:**
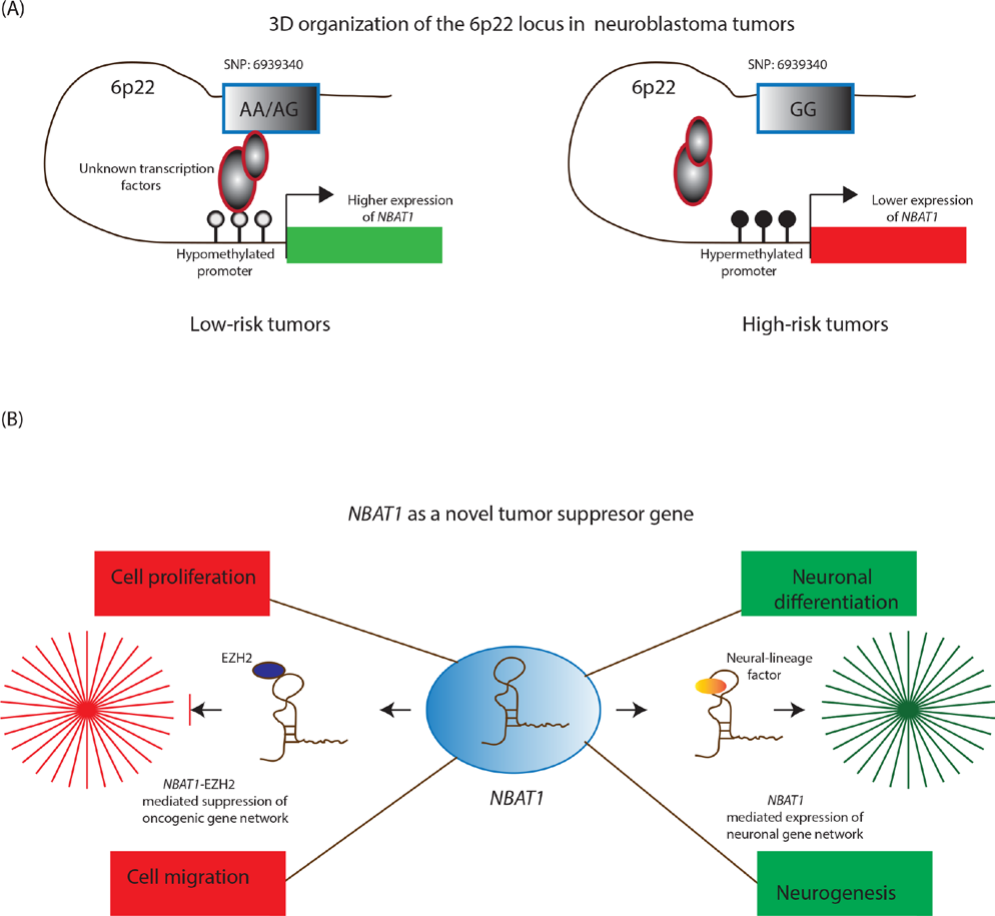
**A.** In low-risk tumors, *NBAT1* is expressed at higher levels due to a three dimensional chromosomal interaction between its hypomethylated promoter, the genotype (A/A; A/G) at SNP: 6939340 and unknown transcription factors. On the contrary, this interaction is disturbed in high-risk patients due to hypermethylation of the *NBAT1* promoter and the presence of high-risk associated genotype (G/G) at the SNP: 6939340. **B.** *NBAT1* is a tumor suppressor lncRNA and by interacting with chromatin remodeling protein EZH2, it controls tumor progression through suppressing oncogenic networks that drive proliferation and invasion of neuroblastoma cells. In addition, it drives neuroblastoma cells towards neural differentiation by suppressing the expression of *NRSF/REST*, which represses neural differentiation program in non-neuronal cells.

The study by *Pandey et al* suggests, there is an intricate interplay of multiple factors that underlay the differential expression of *NBAT1* in NB tumours (Figure 2A) [17]. *In vitro* experiments show that the region harbouring the risk-associated SNP possesses an enhancer like property, with the G/G genotype showing less enhancer activity compared to the A/A genotype. Furthermore, NB cells with the risk-associated G/G genotype showed decreased interaction between the putative enhancer and the *NBAT1* promoter, along with lower levels of enhancer specific chromatin marks at the SNP. The functional association of the SNP genotype and *NBAT1* expression is further substantiated by the observation that expression of this lncRNA is significantly lower in patients with the risk-associated G/G genotype. In addition to these genetic features, the authors found an epigenetic connection between *NBAT1* promoter methylation and its expression. The promoter of this lncRNA is hypermethylated in high-risk NBs when compared to low-risk patients. The reactivation of *NBAT1* upon exposure to DNA demethylating agent 5Aza-2 deoxycytidine suggests that epigenetic-based treatments could be employed for the treatment of high-risk NB patients.

Functional characterization of *NBAT1* in cellular and mouse based model systems illustrated distinct roles of this lncRNA during NB progression. It regulates the proliferative and migratory properties of NB cells through recruitment and targeting of EZH2 (a PRC2 member) to the gene promoters of pro-tumor genes such as *SOX9*, *VCAN* and *OSMR.* EZH2 mediated regulation of these critical gene networks support the role of *NBAT1* as an important tumour-suppressor lncRNA in NBs (Figure 2B) [17]. Taking these observations into account, the following molecular situation can be visualized in high-risk patients. Higher levels of *NBAT1* in low-risk tumours enable its interaction with EZH2 and subsequent recruitment to the *NBAT1* target gene promoters. This in turn leads to H3K27me3 modification of the target gene promoters and gene silencing. On the other hand, lower levels of *NBAT1* in high-risk NBs cause perturbation *of NBAT1/EZH2* mediated gene networks. This perturbation results in an increased proliferation and migration of NB cells due to changes in the chromatin structure of pro-tumour gene promoters. These observations project *NBAT1* as a strong marker in risk-assessment of NB patients and provide significant mechanistic insights into the functional role of *NBAT1* in NB pathogenesis.

#### Other examples of LncRNAs in NB pathogenesis

In addition to the examples discussed above, there are many other lncRNAs that have been observed to be deregulated in NBs and map to regions that do not contain any apparent chromosomal aberrations. For example, a transcriptome profiling of 15 NB tumors has revealed 24 unannotated lncRNAs, which are differentially expressed between low and high-risk neuroblastomas [17]. An interesting feature of these expression signatures is their chromosomal location. The majority of these lncRNAs are present at the chromosomal regions known to be genetically stable in NB patients. Therefore, these lncRNAs should be evaluated for their clinical significance in larger patient cohorts to check whether these expression signatures, similar to protein coding genes, could act as indicators of clinical status of NB patients. In addition, functional characterization of the 24 unannotated lncRNAs could reveal their plausible roles in NB pathogenesis.

*CAI2* (*CDKN2A*/*ARF* Intron 2 lncRNA) is another neuroblastoma associated lncRNA located at the chromosomal locus 9p21, which harbors the well-known tumor suppressor genes *CDKN2A* and *ARF* [57]. *CAI2* is a single exon containing polyadenylated transcript. Its promoter maps to exon γ in *p16* gene. It has five 5 ORFs and the largest ORF is predicted to encode 107 amino acid protein. Interestingly, *CDKN2A* and *ARF* genes, which are normally repressed in the majority of tumors, are upregulated in NBs along with *CAI2* lncRNA and their higher expression correlated with a poor clinical outcome. Like *CDKN2A* and *ARF, CAI2* higher expression is significantly associated with a poor clinical outcome in NBs. *CAI2* is differentially expressed in a stage and age dependent manner and its higher expression in NB patients can independently predict event fee survival. Its clinical outcome has been investigated using a small group of patients and extending clinical studies to larger tumour cohorts will strengthen its clinical significance. In addition, it is unclear how higher expression of *CAI2* induces neuroblastoma pathogenesis. Hence further functional studies in cell culture and xenografts would be necessary to understand its role in NB pathogenesis.

By interfering with the activity of *MYCN*, a microarray-based study has characterized 6 *MYCN* regulated lncRNAs with a functional role in NB pathogenesis [58]. ChIP and luciferase based assays demonstrated that MYCN binds directly to the *linc00467* promoter and represses its promoter activity. Downregulation of *linc00467* in NB cells led to reduced cell viability and increase in apoptosis via activation of the tumor suppressor gene *DKK1*, suggesting that *linc00467* plays an important role in tumorigenesis through reducing the activity of *DKK1*. Although, it regulates cell viability and apoptosis in NB cells, its prognostic value in NB risk assessment is unclear.

*MYCN*, apart from directly regulating lncRNAs expression, it also enhances the expression of lncRNAs through the activation of chromatin remodeling proteins. In a recent report by *Tee et al* have shown that *MYCN* activates cancer–associated lncRNA *MALAT1* through one of its direct target genes *JMJD1A*, which is an H3K9-specific histone demethylase [59]. Thus increased *MYCN* expression in high-risk NB tumors may lead to higher JMJD1A levels, which in turn activates *MALAT1* via demethylating histone H3K9me3 modification at the *MALAT1* gene promoter. *MALAT1* has been shown to promote cell proliferation, cell migration and cell invasion of neuroblastoma cell lines, which is consistent with the metastatic property of *MALAT1* implicated in lung cancer. Loss of these properties in the small molecule JMJD1A inhibitor DMOG treated NB cell lines, suggests that *MYCN* target genes could be used as potential therapeutic targets in the treatment of neuroblastoma.

#### LncRNAs and the differentiation of the neural crest cells

A few subtypes of NB tumours show a unique characteristic of spontaneous regression, not observed in many other cancers. This ability to regress is believed to occur in part through neuronal differentiation and this claim is further strengthened by the use of RA as a routine drug for high-risk NB treatment. Therefore, understanding the pathways/signalling molecules associated with differentiation of neural precursor cells could increase our knowledge about spontaneous tumour regression in NB tumours.

The process of lineage specific differentiation to mature cell types is a tightly regulated event, involving dynamic expression of genes in a spatio-temporal manner. Therefore, it is important to understand these subtle changes in gene expressions and investigate regulators that could control these events. Previous evidence implicated many smaller species of noncoding RNAs (microRNAs) in the control of NB cell differentiation [60]. Recent evidence from next generation sequencing technologies has identified lncRNAs as molecules displaying dynamic expression during lineage commitment of undifferentiated embryonic cells to specific cell types. Their dynamic expression can be extrapolated as lncRNAs being one of the major regulators involved in fine-tuning of the gene expression essential for the lineage determination. For example, a recent investigation has identified 934 lncRNAs as differentially expressed between human ES cells and differentiated neural progenitor cells, and one of the differentially expressed lncRNAs *RMST* has been shown to play a crucial role in neurogenesis [61]. *RMST* interacts with *SOX2*, a known regulator of neuronal fate, and co-regulates *SOX2* downstream genes by influencing the binding of *SOX2* at the target neurogenic gene promoters essential for neuronal differentiation [62]. From the recent literature it is evident that more and more lncRNAs have been shown to play a crucial role in neurogenesis. These studies implicating lncRNAs in nervous system development are of great importance from a NB perspective as these tumours involve perturbed differentiation of neural precursor cells.

In one such example, a highly conserved lncRNA, *Paupar* has been discovered as a differentially expressed transcript during the course of mouse neuronal differentiation [63]. It is a 3.48 kb chromatin associated transcript, located upstream of known neural transcription factor *Pax6. Paupar* mediates a complex transcriptional regulation, including *cis* regulation of upstream gene *Pax6*, and *Pax6* dependent and independent trans functions at other gene regulatory regions. This global targeting of *Paupar* is mediated by its interaction with neural transcriptional factor *Pax6*. Though this RNA has been investigated extensively for its mode of action during neurogenesis, there is no evidence suggesting clinical or functional roles of its human homologue in NB disease development.

In another interesting study by *Pandey et al* has implicated a lncRNA *NBAT1* with a functional role in neuronal differentiation using the SH-SY5Y NB cell line as a model system [17]. SH-SY5Y NB cells have the potential to differentiate into neurons upon RA treatment. *NBAT1* expression is induced during the course of RA induced neural differentiation and its upregulation is required for expression of key neuronal genes required for proper neuronal differentiation. Depletion of *NBAT1* results in poor differentiation of NB cell lines. *NBAT1* controls the neural transcriptional programme by suppressing the negative regulator of differentiation, *NRSF/REST* by associating with an unknown neural lineage specific factor (Figure 2B). These observations hold a greater significance from a clinical perspective, as neural crest cells in high-risk NB cells are known to show poor differentiation. Moreover, overexpression of *NBAT1* in high-risk NB cell lines is able to induce differentiation of tumour cells, thus underscoring its requirement for efficient differentiation of neural precursors in NBs.

The common theme appearing from these studies is that lncRNAs play a greater role in neurogenesis by associating with known (such as *SOX2* or *PAX6*) or unknown transcription factors and regulate the differentiation programs by facilitating the binding of such factors to their target genes promoters. Importantly, these observations support lncRNA molecules as potential drug targets for treating high-risk NB patients with impaired neuronal differentiation.

#### LncRNAs as future tools for diagnosis and treatment of neuroblastomas

Highly tissue- and developmental stage-specific expression patterns along with aberrant expression profiles in cancers are the features that make lncRNAs good candidates for disease diagnosis and prognosis. Interestingly, lncRNAs such as *PCA3* are already being used for diagnostic purposes. It is the first lncRNA to be approved by FDA (Food and drug administration) for usage as a biomarker in urine of prostate cancer patients [64]. The list of lncRNAs which are proposed as biomarkers for cancer diagnosis and prognosis is very long and includes some very well characterized lncRNAs such as *HOTAIR*, *MALAT1*, *HULC* and *ANRIL* [65–69].

In NB tumours identification of additional prognostic biomarkers is greatly warranted due to a high degree of heterogeneity amongst tumour subtypes and their respective clinical outcomes. As discussed earlier, many lncRNA expression profiles could serve as independent biomarkers for NB diagnosis. However, to pass the test for their clinical usage, their proposed diagnostic and clinical functions must be evaluated in larger and varied datasets. Some of the lncRNAs such as *NBAT1* and *lncUSMycN* have been investigated for their clinical significance in larger datasets and can be considered for future clinical usage in risk assessment of NB patients [17, 30, 40]. Importantly, *NBAT1* is an independent predictor of event free survival amongst high-risk patients. Also, *NBAT1* shows lower expression in all high-risk tumours including both the MYCN and 11q-subtypes, which constitute a major fraction of high-risk NB tumours. This in particular increases its scope for prognostic usage in the majority of NB subtypes for risk assessment. In addition, clinical and functional investigations of *NBAT1* lncRNA indicate that it could be a potential therapeutic target. Activation of *NBAT1* upon treatment with a demethylating agent in NB cells indicate that, in addition to RA, epigenetic-based intervention can be considered in the treatment of high-risk NBs. Combined use of RA and epigenetic based intervention could promote efficient differentiation of undifferentiated stem cells into neurons, thereby decreasing the tumor cell population.

The clinical and functional relevance of lncRNAs discussed above in NB pathogenesis have to be studied in greater detail for them to be used on par with their protein-coding counterparts in the clinic. Investigating their expression patterns in body fluids (such as blood, saliva and urine) that are routinely used in clinic for diagnostic purposes would expedite the diagnosis. Being relatively stable molecules that can be found circulating in body fluids, their clinical correlation with patients risk condition could pitch them for usage as diagnostic molecules in the clinic.

### Conclusion and future perspective

The explosion of data from genomics and RNAomics studies over the last decade, both in normal and disease conditions, has brought lncRNAs into focus. The significance of these regulatory molecules has remained underrated as well as less well understood in NB tumours. In this review, we provide an overview of lncRNAs, whose role in NB tumours is beginning to be uncovered. These discussed examples clearly emphasize the clinical and biological contribution of lncRNAs towards understanding the underlying biology of NB tumours. It would be interesting to focus future research on involving larger sets of patient cohorts. This would allow us to identify new as well as study the existing lncRNAs for their use in clinics. Furthermore, functional studies using cell and mouse based approaches may highlight pathways with important roles in the process of tumorigenesis.

## References

[1] Kamijo T, Nakagawara A (2012). Molecular and genetic bases of neuroblastoma. International journal of clinical oncology.

[2] Brodeur GM (2003). Neuroblastoma: biological insights into a clinical enigma. Nature reviews Cancer.

[3] Cohn SL, Pearson AD, London WB, Monclair T, Ambros PF, Brodeur GM, Faldum A, Hero B, Iehara T, Machin D, Mosseri V, Simon T, Garaventa A, Castel V, Matthay KK, Force IT (2009). The International Neuroblastoma Risk Group (INRG) classification system: an INRG Task Force report. Journal of clinical oncology : official journal of the American Society of Clinical Oncology.

[4] Monclair T, Brodeur GM, Ambros PF, Brisse HJ, Cecchetto G, Holmes K, Kaneko M, London WB, Matthay KK, Nuchtern JG, von Schweinitz D, Simon T, Cohn SL, Pearson AD, Force IT (2009). The International Neuroblastoma Risk Group (INRG) staging system: an INRG Task Force report. Journal of clinical oncology : official journal of the American Society of Clinical Oncology.

[5] Brodeur GM, Seeger RC, Schwab M, Varmus HE, Bishop JM (1984). Amplification of N-myc in untreated human neuroblastomas correlates with advanced disease stage. Science.

[6] Bagatell R, Beck-Popovic M, London WB, Zhang Y, Pearson AD, Matthay KK, Monclair T, Ambros PF, Cohn SL, International Neuroblastoma Risk G (2009). Significance of MYCN amplification in international neuroblastoma staging system stage 1 and 2 neuroblastoma: a report from the International Neuroblastoma Risk Group database. Journal of clinical oncology : official journal of the American Society of Clinical Oncology.

[7] Bordow SB, Norris MD, Haber PS, Marshall GM, Haber M (1998). Prognostic significance of MYCN oncogene expression in childhood neuroblastoma. Journal of clinical oncology : official journal of the American Society of Clinical Oncology.

[8] Schwab M (1993). Amplification of N-myc as a prognostic marker for patients with neuroblastoma. Seminars in cancer biology.

[9] Caron H (1995). Allelic loss of chromosome 1 and additional chromosome 17 material are both unfavourable prognostic markers in neuroblastoma. Medical and pediatric oncology.

[10] Schleiermacher G, Mosseri V, London WB, Maris JM, Brodeur GM, Attiyeh E, Haber M, Khan J, Nakagawara A, Speleman F, Noguera R, Tonini GP, Fischer M, Ambros I, Monclair T, Matthay KK (2012). Segmental chromosomal alterations have prognostic impact in neuroblastoma: a report from the INRG project. British journal of cancer.

[11] Maris JM, Matthay KK (1999). Molecular biology of neuroblastoma. Journal of clinical oncology : official journal of the American Society of Clinical Oncology.

[12] Maris JM, White PS, Beltinger CP, Sulman EP, Castleberry RP, Shuster JJ, Look AT, Brodeur GM (1995). Significance of chromosome 1p loss of heterozygosity in neuroblastoma. Cancer research.

[13] Theissen J, Oberthuer A, Hombach A, Volland R, Hertwig F, Fischer M, Spitz R, Zapatka M, Brors B, Ortmann M, Simon T, Hero B, Berthold F (2014). Chromosome 17/17q gain and unaltered profiles in high resolution array-CGH are prognostically informative in neuroblastoma. Genes, chromosomes & cancer.

[14] Diskin SJ, Capasso M, Schnepp RW, Cole KA, Attiyeh EF, Hou C, Diamond M, Carpenter EL, Winter C, Lee H, Jagannathan J, Latorre V, Iolascon A, Hakonarson H, Devoto M, Maris JM (2012). Common variation at 6q16 within HACE1 and LIN28B influences susceptibility to neuroblastoma. Nature genetics.

[15] Maris JM, Mosse YP, Bradfield JP, Hou C, Monni S, Scott RH, Asgharzadeh S, Attiyeh EF, Diskin SJ, Laudenslager M, Winter C, Cole KA, Glessner JT, Kim C, Frackelton EC, Casalunovo T (2008). Chromosome 6p22 locus associated with clinically aggressive neuroblastoma. The New England journal of medicine.

[16] Molenaar JJ, Domingo-Fernandez R, Ebus ME, Lindner S, Koster J, Drabek K, Mestdagh P, van Sluis P, Valentijn LJ, van Nes J, Broekmans M, Haneveld F, Volckmann R, Bray I, Heukamp L, Sprussel A (2012). LIN28B induces neuroblastoma and enhances MYCN levels via let-7 suppression. Nature genetics.

[17] Pandey GK, Mitra S, Subhash S, Hertwig F, Kanduri M, Mishra K, Fransson S, Ganeshram A, Mondal T, Bandaru S, Ostensson M, Akyurek LM, Abrahamsson J, Pfeifer S, Larsson E, Shi L (2014). The risk-associated long noncoding RNA NBAT-1 controls neuroblastoma progression by regulating cell proliferation and neuronal differentiation. Cancer cell.

[18] Maris JM (2010). Recent advances in neuroblastoma. The New England journal of medicine.

[19] Morris KV, Mattick JS (2014). The rise of regulatory RNA. Nature reviews Genetics.

[20] Mercer TR, Dinger ME, Mattick JS (2009). Long non-coding RNAs: insights into functions. Nature reviews Genetics.

[21] Batista PJ, Chang HY (2013). Long noncoding RNAs: cellular address codes in development and disease. Cell.

[22] Kanduri C (2011). Long noncoding RNA and epigenomics. Advances in experimental medicine and biology.

[23] Mohammad F, Mondal T, Kanduri C (2009). Epigenetics of imprinted long noncoding RNAs. Epigenetics : official journal of the DNA Methylation Society.

[24] Whitehead J, Pandey GK, Kanduri C (2009). Regulation of the mammalian epigenome by long noncoding RNAs. Biochimica et biophysica acta.

[25] Ohms S, Rangasamy D (2014). Silencing of LINE-1 retrotransposons contributes to variation in small noncoding RNA expression in human cancer cells. Oncotarget.

[26] Prensner JR, Chinnaiyan AM (2011). The emergence of lncRNAs in cancer biology. Cancer discovery.

[27] Spizzo R, Almeida MI, Colombatti A, Calin GA (2012). Long non-coding RNAs and cancer: a new frontier of translational research?. Oncogene.

[28] Taft RJ, Pang KC, Mercer TR, Dinger M, Mattick JS (2010). Non-coding RNAs: regulators of disease. The Journal of pathology.

[29] Xue Y, Ma G, Zhang Z, Hua Q, Chu H, Tong N, Yuan L, Qin C, Yin C, Zhang Z, Wang M (2015). A novel antisense long noncoding RNA regulates the expression of MDC1 in bladder cancer. Oncotarget.

[30] Pandey GK, Kanduri C (2015). Fighting Neuroblastomas with NBAT1. Oncoscience.

[31] Liu B, Sun L, Liu Q, Gong C, Yao Y, Lv X, Lin L, Yao H, Su F, Li D, Zeng M, Song E (2015). A cytoplasmic NF-kappaB interacting long noncoding RNA blocks IkappaB phosphorylation and suppresses breast cancer metastasis. Cancer cell.

[32] Lee KS, Park JL, Lee K, Richardson LE, Johnson BH, Lee HS, Lee JS, Kim SB, Kwon OH, Song KS, Kim YS, Ashktorab H, Smoot DT, Jeon SH, Kim SY, Lee YS (2014). nc886, a non-coding RNA of anti-proliferative role, is suppressed by CpG DNA methylation in human gastric cancer. Oncotarget.

[33] Lee HS, Lee K, Jang HJ, Lee GK, Park JL, Kim SY, Kim SB, Johnson BH, Zo JI, Lee JS, Lee YS (2014). Epigenetic silencing of the non-coding RNA nc886 provokes oncogenes during human esophageal tumorigenesis. Oncotarget.

[34] Wu Y, Liu H, Shi X, Yao Y, Yang W, Song Y (2015). The long non-coding RNA HNF1A-AS1 regulates proliferation and metastasis in lung adenocarcinoma. Oncotarget.

[35] Mazar J, Zhao W, Khalil AM, Lee B, Shelley J, Govindarajan SS, Yamamoto F, Ratnam M, Aftab MN, Collins S, Finck BN, Han X, Mattick JS, Dinger ME, Perera RJ (2014). The functional characterization of long noncoding RNA SPRY4-IT1 in human melanoma cells. Oncotarget.

[36] Guo H, Zhang X, Dong R, Liu X, Li Y, Lu S, Xu L, Wang Y, Wang X, Hou D, Wei JJ, Shao C, Liu Z (2014). Integrated analysis of long noncoding RNAs and mRNAs reveals their potential roles in the pathogenesis of uterine leiomyomas. Oncotarget.

[37] Li H, Yu B, Li J, Su L, Yan M, Zhu Z, Liu B (2014). Overexpression of lncRNA H19 enhances carcinogenesis and metastasis of gastric cancer. Oncotarget.

[38] Buechner J, Einvik C (2012). N-myc and noncoding RNAs in neuroblastoma. Molecular cancer research : MCR.

[39] Castelnuovo M, Massone S, Tasso R, Fiorino G, Gatti M, Robello M, Gatta E, Berger A, Strub K, Florio T, Dieci G, Cancedda R, Pagano A (2010). An Alu-like RNA promotes cell differentiation and reduces malignancy of human neuroblastoma cells. FASEB journal : official publication of the Federation of American Societies for Experimental Biology.

[40] Liu PY, Erriquez D, Marshall GM, Tee AE, Polly P, Wong M, Liu B, Bell JL, Zhang XD, Milazzo G, Cheung BB, Fox A, Swarbrick A, Huttelmaier S, Kavallaris M, Perini G (2014). Effects of a novel long noncoding RNA, lncUSMycN, on N-Myc expression and neuroblastoma progression. Journal of the National Cancer Institute.

[41] Iyer MK, Niknafs YS, Malik R, Singhal U, Sahu A, Hosono Y, Barrette TR, Prensner JR, Evans JR, Zhao S, Poliakov A, Cao X, Dhanasekaran SM, Wu YM, Robinson DR, Beer DG (2015). The landscape of long noncoding RNAs in the human transcriptome. Nature genetics.

[42] Pandey RR, Mondal T, Mohammad F, Enroth S, Redrup L, Komorowski J, Nagano T, Mancini-Dinardo D, Kanduri C (2008). Kcnq1ot1 antisense noncoding RNA mediates lineage-specific transcriptional silencing through chromatin-level regulation. Molecular cell.

[43] Gibb EA, Brown CJ, Lam WL (2011). The functional role of long non-coding RNA in human carcinomas. Molecular cancer.

[44] Bejerano G, Pheasant M, Makunin I, Stephen S, Kent WJ, Mattick JS, Haussler D (2004). Ultraconserved elements in the human genome. Science.

[45] Calin GA, Liu CG, Ferracin M, Hyslop T, Spizzo R, Sevignani C, Fabbri M, Cimmino A, Lee EJ, Wojcik SE, Shimizu M, Tili E, Rossi S, Taccioli C, Pichiorri F, Liu X (2007). Ultraconserved regions encoding ncRNAs are altered in human leukemias and carcinomas. Cancer cell.

[46] Scaruffi P, Stigliani S, Moretti S, Coco S, De Vecchi C, Valdora F, Garaventa A, Bonassi S, Tonini GP (2009). Transcribed-Ultra Conserved Region expression is associated with outcome in high-risk neuroblastoma. BMC cancer.

[47] Mestdagh P, Fredlund E, Pattyn F, Rihani A, Van Maerken T, Vermeulen J, Kumps C, Menten B, De Preter K, Schramm A, Schulte J, Noguera R, Schleiermacher G, Janoueix-Lerosey I, Laureys G, Powel R (2010). An integrative genomics screen uncovers ncRNA T-UCR functions in neuroblastoma tumours. Oncogene.

[48] Watters KM, Bryan K, Foley NH, Meehan M, Stallings RL (2013). Expressional alterations in functional ultra-conserved non-coding RNAs in response to all-trans retinoic acid—induced differentiation in neuroblastoma cells. BMC cancer.

[49] Bown N, Cotterill S, Lastowska M, O'Neill S, Pearson AD, Plantaz D, Meddeb M, Danglot G, Brinkschmidt C, Christiansen H, Laureys G, Speleman F, Nicholson J, Bernheim A, Betts DR, Vandesompele J (1999). Gain of chromosome arm 17q and adverse outcome in patients with neuroblastoma. The New England journal of medicine.

[50] Azuhata T, Scott D, Takamizawa S, Wen J, Davidoff A, Fukuzawa M, Sandler A (2001). The inhibitor of apoptosis protein survivin is associated with high-risk behavior of neuroblastoma. Journal of pediatric surgery.

[51] Saito-Ohara F, Imoto I, Inoue J, Hosoi H, Nakagawara A, Sugimoto T, Inazawa J (2003). PPM1D is a potential target for 17q gain in neuroblastoma. Cancer research.

[52] Yu M, Ohira M, Li Y, Niizuma H, Oo ML, Zhu Y, Ozaki T, Isogai E, Nakamura Y, Koda T, Oba S, Yu B, Nakagawara A (2009). High expression of ncRAN, a novel non-coding RNA mapped to chromosome 17q25.1, is associated with poor prognosis in neuroblastoma. International journal of oncology.

[53] Seeger RC, Brodeur GM, Sather H, Dalton A, Siegel SE, Wong KY, Hammond D (1985). Association of multiple copies of the N-myc oncogene with rapid progression of neuroblastomas. The New England journal of medicine.

[54] Tseng YY, Moriarity BS, Gong W, Akiyama R, Tiwari A, Kawakami H, Ronning P, Reuland B, Guenther K, Beadnell TC, Essig J, Otto GM, O'Sullivan MG, Largaespada DA, Schwertfeger KL, Marahrens Y (2014). PVT1 dependence in cancer with MYC copy-number increase. Nature.

[55] Attiyeh EF, London WB, Mosse YP, Wang Q, Winter C, Khazi D, McGrady PW, Seeger RC, Look AT, Shimada H, Brodeur GM, Cohn SL, Matthay KK, Maris JM, Children's Oncology G (2005). Chromosome 1p and 11q deletions and outcome in neuroblastoma. The New England journal of medicine.

[56] Caren H, Kryh H, Nethander M, Sjoberg RM, Trager C, Nilsson S, Abrahamsson J, Kogner P, Martinsson T (2010). High-risk neuroblastoma tumors with 11q-deletion display a poor prognostic, chromosome instability phenotype with later onset. Proceedings of the National Academy of Sciences of the United States of America.

[57] Barnhill LM, Williams RT, Cohen O, Kim Y, Batova A, Mielke JA, Messer K, Pu M, Bao L, Yu AL, Diccianni MB (2014). High expression of CAI2, a 9p21-embedded long noncoding RNA, contributes to advanced-stage neuroblastoma. Cancer research.

[58] Atmadibrata B, Liu PY, Sokolowski N, Zhang L, Wong M, Tee AE, Marshall GM, Liu T (2014). The novel long noncoding RNA linc00467 promotes cell survival but is down-regulated by N-Myc. PloS one.

[59] Tee AE, Ling D, Nelson C, Atmadibrata B, Dinger ME, Xu N, Mizukami T, Liu PY, Liu B, Cheung B, Pasquier E, Haber M, Norris MD, Suzuki T, Marshall GM, Liu T (2014). The histone demethylase JMJD1A induces cell migration and invasion by up-regulating the expression of the long noncoding RNA MALAT1. Oncotarget.

[60] Zhao Z, Ma X, Hsiao TH, Lin G, Kosti A, Yu X, Suresh U, Chen Y, Tomlinson GE, Pertsemlidis A, Du L (2014). A high-content morphological screen identifies novel microRNAs that regulate neuroblastoma cell differentiation. Oncotarget.

[61] Ng SY, Johnson R, Stanton LW (2012). Human long non-coding RNAs promote pluripotency and neuronal differentiation by association with chromatin modifiers and transcription factors. The EMBO journal.

[62] Ng SY, Bogu GK, Soh BS, Stanton LW (2013). The long noncoding RNA RMST interacts with SOX2 to regulate neurogenesis. Molecular cell.

[63] Vance KW, Sansom SN, Lee S, Chalei V, Kong L, Cooper SE, Oliver PL, Ponting CP (2014). The long non-coding RNA Paupar regulates the expression of both local and distal genes. The EMBO journal.

[64] de Kok JB, Verhaegh GW, Roelofs RW, Hessels D, Kiemeney LA, Aalders TW, Swinkels DW, Schalken JA (2002). DD3(PCA3), a very sensitive and specific marker to detect prostate tumors. Cancer research.

[65] Vitiello M, Tuccoli A, Poliseno L (2014). Long non-coding RNAs in cancer: implications for personalized therapy. Cellular oncology.

[66] Wang F, Ren S, Chen R, Lu J, Shi X, Zhu Y, Zhang W, Jing T, Zhang C, Shen J, Xu C, Wang H, Wang H, Wang Y, Liu B, Li Y (2014). Development and prospective multicenter evaluation of the long noncoding RNA MALAT-1 as a diagnostic urinary biomarker for prostate cancer. Oncotarget.

[67] Zhang EB, Kong R, Yin DD, You LH, Sun M, Han L, Xu TP, Xia R, Yang JS, De W, Chen J (2014). Long noncoding RNA ANRIL indicates a poor prognosis of gastric cancer and promotes tumor growth by epigenetically silencing of miR-99a/miR-449a. Oncotarget.

[68] Hu Y, Chen HY, Yu CY, Xu J, Wang JL, Qian J, Zhang X, Fang JY (2014). A long non-coding RNA signature to improve prognosis prediction of colorectal cancer. Oncotarget.

[69] Crea F, Watahiki A, Quagliata L, Xue H, Pikor L, Parolia A, Wang Y, Lin D, Lam WL, Farrar WL, Isogai T, Morant R, Castori-Eppenberger S, Chi KN, Wang Y, Helgason CD (2014). Identification of a long non-coding RNA as a novel biomarker and potential therapeutic target for metastatic prostate cancer. Oncotarget.

